# Supplementary material for: Trends in Survival and Mortality of “Early” Metastatic Breast Cancer in Northern Italy Following the Introduction of Targeted Therapies
Source: Cancers (Basel). 2025 Dec 29;18(1):108. doi: 10.3390/cancers18010108 (PMC12784658; doi:10.3390/cancers18010108)
Supplement: Supplementary file 1 [file cancers-18-00108-s001.zip › cancers-4023339-supplementary.pdf]

Table S1. Emerging Drugs and Nano-based Delivery Strategies in Metastatic Breast Cancer

Therapeutic category	Key agents	Mechanism / Innovation	Evidence in MBC
**Antibody–Drug Conjugates (ADCs)**	T-DXd, SG, T-DM1	Targeted cytotoxic delivery; high DAR; by-stander effect	Major OS/PFS gains in HER2+ and HER2-low
**Nano-based delivery systems**	Liposomal doxorubicin; nab-paclitaxel; polymeric/lipid nanoparticles	Improved bioavailability, reduced toxicity	Benefit in TNBC; ongoing trials
**HER2 novel agents**	Tucatinib; margetuximab; bispecific antibodies	Selective HER2 blockade; CNS penetration	Survival and CNS control improvement
**Endocrine next-generation agents**	Oral SERDs; ER-PROTACs	Overcomes mutant resistance	PFS benefit
**CDK4/6 inhibitors**	Ribociclib; palbociclib; abemaciclib	CDK4/6 blockade	Consistent OS benefit
**PI3K–AKT–mTOR inhibitors**	Alpelisib; capivasertib; everolimus	Target resistance pathways	Improved PFS in altered tumors
**Immunotherapy**	Pembrolizumab; atezolizumab	Immune checkpoint inhibition	OS benefit in PD-L1+ TNBC
**Radiopharmaceuticals**	HER2-radio-conjugates	Selective irradiation	Early-phase activity
**Advanced nanotechnologies**	Exosomes; biomimetic nanoparticles	Ultra-selective targeting	Preclinical favorable data
